# Cardiovascular MRI Compared to Echocardiography to Identify Cardioaortic Sources of Ischemic Stroke: A Systematic Review and Meta-Analysis

**DOI:** 10.3389/fneur.2021.699838

**Published:** 2021-07-30

**Authors:** Thomas R. Meinel, Angela Eggimann, Kristina Brignoli, Kerstin Wustmann, Eric Buffle, Felix G. Meinel, Jan F. Scheitz, Christian H. Nolte, Christoph Gräni, Urs Fischer, Johannes Kaesmacher, David J. Seiffge, Christian Seiler, Simon Jung

**Affiliations:** ^1^Department of Neurology, Inselspital, Bern University Hospital, and University of Bern, Bern, Switzerland; ^2^Department of Cardiology, Inselspital Bern, Bern University Hospital, and University of Bern, Bern, Switzerland; ^3^Institute of Diagnostic and Interventional Radiology, Pediatric Radiology and Neuroradiology, Rostock University Medical Center, Rostock, Germany; ^4^Klinik und Hochschulambulanz für Neurologie, Charité-Universitätsmedizin Berlin, Berlin, Germany; ^5^Klinik für Neurologie, Berlin Institute of Health, Berlin, Germany; ^6^German Centre for Cardiovascular Research, Deutsches Zentrum für Herz-Kreislauf-Forschung, Berlin, Germany; ^7^Institute of Diagnostic and Interventional Neuroradiology, Inselspital, Bern University Hospital, Bern, Switzerland

**Keywords:** cardiac MRI, echocardiography, ischemic stroke, diagnostic work up, cardioaortic embolism

## Abstract

**Background:** To compare the diagnostic yield of echocardiography and cardiovascular MRI (CMR) to detect structural sources of embolism, in patients with ischemic stroke with a secondary analysis of non-stroke populations.

**Methods and Results:** We searched MEDLINE/Embase (from 01.01.2000 to 24.04.2021) for studies including CMR to assess prespecified sources of embolism. Comparison included transthoracic and/or transesophageal echocardiography. Two authors independently screened studies, extracted data and assessed bias using the QUADAS-2 tool. Estimates of diagnostic yield were reported and pooled. Twenty-seven studies with 2,525 patients were included in a study-level analysis. Most studies had moderate to high risk of bias. Persistent foramen ovale, complex aortic plaques, left ventricular and left atrial thrombus were the most common pathologies. There was no difference in the yield of left ventricular thrombus detection between both modalities for stroke populations (4 studies), but an increased yield of CMR in non-stroke populations (28.1 vs. 16.0%, *P* < 0.001, 10 studies). The diagnostic yield in stroke patients for detection of persistent foramen ovale was lower in CMR compared to transoesophageal echocardiography (29.3 vs. 53.7%, *P* < 0.001, 5 studies). For both echocardiography and CMR the clinical impact of the management consequences derived from many of the diagnostic findings remained undetermined in the identified studies.

**Conclusions:** Echocardiography and CMR seem to have similar diagnostic yield for most cardioaortic sources of embolism except persistent foramen ovale and left ventricular thrombus. Randomized controlled diagnostic trials are necessary to understand the impact on the management and potential clinical benefits of the assessment of structural cardioaortic stroke sources.

**Registration:** PROSPERO: CRD42020158787.

## Introduction

Emboli from the heart and great supraaortic arteries account for about 25% of acute ischemic stroke (AIS) (1). The most frequently used diagnostic tests to identify structural cardioembolic sources (see Table 1 for potential sources) (2) are transthoracic (TTE) or transesophageal echocardiography (TEE). However, the number needed to screen to change management on an evidence-based principle is relatively high (3, 4) and there is a notorious debate about the yield, usefulness and optimal patient selection regarding echocardiography in AIS patients (5–7). Hence, the effectiveness of routine echocardiography in unselected AIS patients to optimize treatment selection for prevention of recurrent cardiovascular events was deemed uncertain by the 2019 update of the guidelines for the management of acute stroke with a moderate evidence level supporting this statement (5).

**Table 1:** Cardioaortic sources of embolism, management implications, and diagnostic tests.

	**Target conditions**	**(Potential) management implications**	**Presumed diagnostic performance**				**Estimated frequency (%) in ischemic stroke patients**	**Prespecified for meta-analysis**	**Comment**
			**CMR**	**TTE**	**TEE**	**CTA**			
High-risk sources of embolism	Left atrial (appendage) thrombus	Anticoagulation, (Left atrial appendage occlusion), Prolonged screening for atrial fibrillation	++	+	+ + +	++	3–16%	Yes	Dense spontaneous echo contrast (SEC) similar to thrombus
	Left ventricular thrombus: - Unrecognized Myocardial Infarction (e.g., hypokinesia, scarring) - Dilated cardiomyopathy	Anticoagulation (Coronary angiography/Bypass) Work-up	+ + +	++ (CE)	++ (CE)	++	1–5% 2–4%	Yes	Risk increases with size, protrusion and mobility
	Valvular vegetations: - Infective endocarditis - non-infective/marantic	Antibiotics, (surgery) Anticoagulation, underlying condition	?	++	+ + +	?	3% 1–2% <1%	Yes	Clinical signs including fever, heart murmur, etc. Risk increases with size and mobility
	Non-thrombotic masses, e.g., Myxoma or other tumors	(Surgery)	+ + +	+	++	++	<1%	Yes	
	Valve thrombosis	Anticoagulation, (surgery)	?	++	+ + +	+	<1%		
	Aortic dissection	(Surgery)	++	-	++	+ + +	<1%		
	Valvular atrial fibrillation due to Mitral Valve Stenosis	Anticoagulation (Vitamin K Antagonists), surveillance (intervention)	+	++	+ + +	+	<1%		
	Persistent foramen ovale and/or Atrial septal aneurysm	(Closure)	+	+ (CE)	+++ (CE)	+	PFO 5–35%, ASA 13%	Yes	Inconclusive evidence for routine closure
Moderate-risk sources of embolism	Complex aortic plaques	Statin (surgery, dual antiplatelet therapy)	++		++	+ + +	5–30%	Yes	Definition unclear (mostly ≥4 mm, ulcerated or free-floating thrombus), optimal management unclear
	LV-aneurysm, transmural scarring	Anticoagulation	+ + +	++	++	++	1%		
	Left ventricular non-compaction and other cardiomyopathies	Heart failure treatment (anticoagulation), ICD-Evaluation	+ + +	++	++	++	<1%		Absolute risk unclear
	Left atrial volume, morphology and function	(Long-term monitoring for atrial fibrillation)	+ + +	+	++	++	20–25%		
	Aortitis	Antiinflammatory treatment	+ + +	+	++	+	<1%		
Uncertain risk	Structural Correlates of atrial dysrhythmias (e.g., atrial fibrosis)	(Prolonged rhythm monitoring, anticoagulation)	+ + +	-	-	-			
	Spontaneous echo contrast “smoke”	(Anticoagulation)	+	-	+ + +	+	2–15%		Higher risk with dense SEC
	High grade valve disease/calcifications	(Surgery, intervention)	++	++	+ + +	++	5%		Risk is very variable and uncertain
	Mitral valve prolapse		+	++	+ + +	+	2–5%		Risk is very variable and uncertain
	Valvular strands		?	+	+ + +	?			Risk is very uncertain
Other Findings Requiring Management changes without clear connection to embolic event	Hypo- and/or Akinesia, coronary artery disease	Coronary angiography/Bypass	+ + +	++	+	++	?		Coronary angiography gold standard for coronary artery disease
	Severely reduced ejection fraction	Work-up, Coronary angiography/Bypass	+ + +	++	+	++	5–10%		
	Aortic aneurysm	Surveillance, surgery	++	+	++	+ + +	?		

*CMR, Cardiovascular MRI; TTE, transthoracic echocardiography; TEE, transesophageal echocardiography; CTA, cardiac CT-Angiography; + + +, presumed diagnostic gold standard with regard to each pathology; ++, reasonable diagnostic performance; +, poor diagnostic performance; ?, unclear diagnostic performance; CE, contrast enhanced; PFO, persistent foramen ovale; ASA, atrial septal aneurysm*.

Cardiovascular magnetic resonance imaging (CMR) has the ability to identify intraventricular thrombi and thrombi in the left atrium (LA) and left atrial appendage (8). Furthermore, ulcerated/complex aortic arch atherosclerotic plaques, left ventricular non-compaction cardiomyopathy, and other cardiac structural abnormalities (i.e., LA fibrosis, inherited cardiomyopathy, infiltrative cardiopathy) are potentially relevant findings on CMR with consequences regarding AIS patient management (9–13). Additionally, CMR offers the ability to detect subendocardial or transmural left ventricular scars from recent or previous myocardial infarction. The latter is important as wall motion abnormalities may promote intracardiac thrombus formation and serve as a cardioembolic source of strokes, and may have long-term prognostic implications (14–16). As outlined in a previous narrative review, CMR may represent a potential diagnostic method in patients after embolic stroke with undetermined etiology or—more precisely—of unknown source (ESUS) although heterogeneity in indication strategies, accessibility and feasibility of CMR have to be taken into account (17).

In view of these observational reports and owing to the lack of randomized data, we conducted a systematic review to report on the diagnostic yield of CMR and echocardiography (TTE and/or TEE) as diagnostic strategy for work-up of structural cardio-aortic sources of embolism in AIS patients.

## Methods

### Registration and Data Availability Statement

Performed according to the Preferred Reporting Items for Systematic Reviews and Meta-analyses guideline for Diagnostic Test Accuracy studies (18). The protocol has been published prior to performing the analysis (PROSPERO:CRD42020158787). Data will be shared upon request from any qualified investigator for the purposes of replicating results.

### Study Eligibility Criteria

Eligible studies included adults (older than 16 years) and all ethnic groups reporting on established, predefined structural high-risk sources embolism in the context of AIS (Table 1) (19, 20). For the primary analysis, only patients in the AIS setting who underwent both, CMR and echocardiography during hospitalization or later, were included. In a secondary analysis, studies in non-stroke populations were included when they reported the predefined high-risk sources. We excluded studies reporting on congenital heart diseases (*post-hoc* exclusion after registration). We included peer-reviewed, original studies only. Since state-of-the-art CMR was used in the last two decades, we only considered publications beyond January 1st, 2000 for analysis. We selected the study with the larger sample size in case of studies with duplicate data. We excluded studies with less than five patients undergoing both diagnostic methods.

#### Data Sources and Searches

We searched PubMed/EMBASE using a predefined search strategy combining MeSH terms and keywords with the concepts of CMR, echocardiography and AIS (see Supplementary Material and Table 2 for full details). We used additional references from papers if relevant information was provided. We included articles published in English, German, French or Spanish between January 1st, 2000 and April 24th, 2021.

**Table 2:** Search strategy.

Concept 1 (cardiac MRI)	“Magnetic Resonance Imaging, Cine”[MeSh] OR cardiac MR^*^[title/abstract] OR CMR[title/abstract] OR ((cardiac[Title/Abstract] OR heart[Title/Abstract]) AND (Magnetic Resonance Imaging^*^[Title/Abstract] OR MRI^*^[Title/Abstract]))
**AND**	
Concept 2 (echocardiography)	“Echocardiography”[MeSh] OR “Echocardiography, Transesophageal”[MeSh] OR echocardi^*^[title/abstract] OR “Cardiac Imaging Techniques”[MeSh]
**AND**	
Concept 3 (ischemic stroke)	“Stroke”[Mesh] OR “Cerebrovascular Disorders”[MeSh] OR “Intracranial Embolism and Thrombosis”[Mesh] OR “stroke”[title/abstract] OR ischemic stroke[Title/Abstract] OR brain infarction^*^[Title/Abstract]
Date filter	“2001/01/01”[PDAT]: “2021/04/24”[PDAT] AND
Further filters	“humans”[MeSH Terms] AND (English[lang] OR French[lang] OR German[lang] OR Spanish[lang]

*Combination of Medical Subject Headings and keywords for the concepts of cardiac MRI, echocardiography and ischemic stroke*.

#### Study Selection

Two independent researchers (TRM and AE) assessed the eligibility based on title and abstract using the covidence online review tool. Full-text manuscripts were obtained for all eligible studies before full-text review. Any differences regarding eligibility were resolved by consensus.

#### Data Collection Process and Data Items

We extracted data on study characteristics (study design, publication year, sample size, target condition) and study population characteristics (country, inclusion/exclusion criteria). We also collected data on CMR and echocardiography techniques, such as magnetic field strength, use of contrast agent, time interval between tests, and rate of pathological findings. We extracted study data on predefined forms using study-specific definitions for the predefined target condition(s).

#### Risk of Bias in Individual and Across Studies

We compared data items, study design strengths and weaknesses. We assessed the risk of bias at the study level using the QUADAS-2 bias assessment tool with study-specific items for studies included in the quantitative analysis.

#### Index Test and Reference Standard, Study Endpoints

Due to the diverse structural sources with differing gold standards for each pathology, we chose diagnostic yield as the primary endpoint. Diagnostic yield was defined as the proportion of patients with a specific pathology on CMR or echocardiography. Sensitivity and specificity of CMR as compared to echocardiography were defined as secondary outcome. For this analysis, CMR was chosen as the index test and echocardiography defined as the reference. Other endpoints reported in qualitative manner included feasibility of CMR, and change of management in AIS patients according to imaging findings.

#### Synthesis of Results and Summary Measures

We used McNemar test for paired data to determine differences in diagnostic yield using Stata (StataCorp. 2019. Stata Statistical Software: Release 16. College Station, TX: StataCorp LLC). Measures of diagnostic accuracy, sensitivity and specificity of CMR vs. echocardiography were calculated for all studies based on reported cases with the identified pathology. Forest plots were generated using Review Manager version 5.3 (RevMan, Copenhagen: The Nordic Cochrane Centre, The Cochrane Collaboration, 2014).

## Results

**Figure 1:**
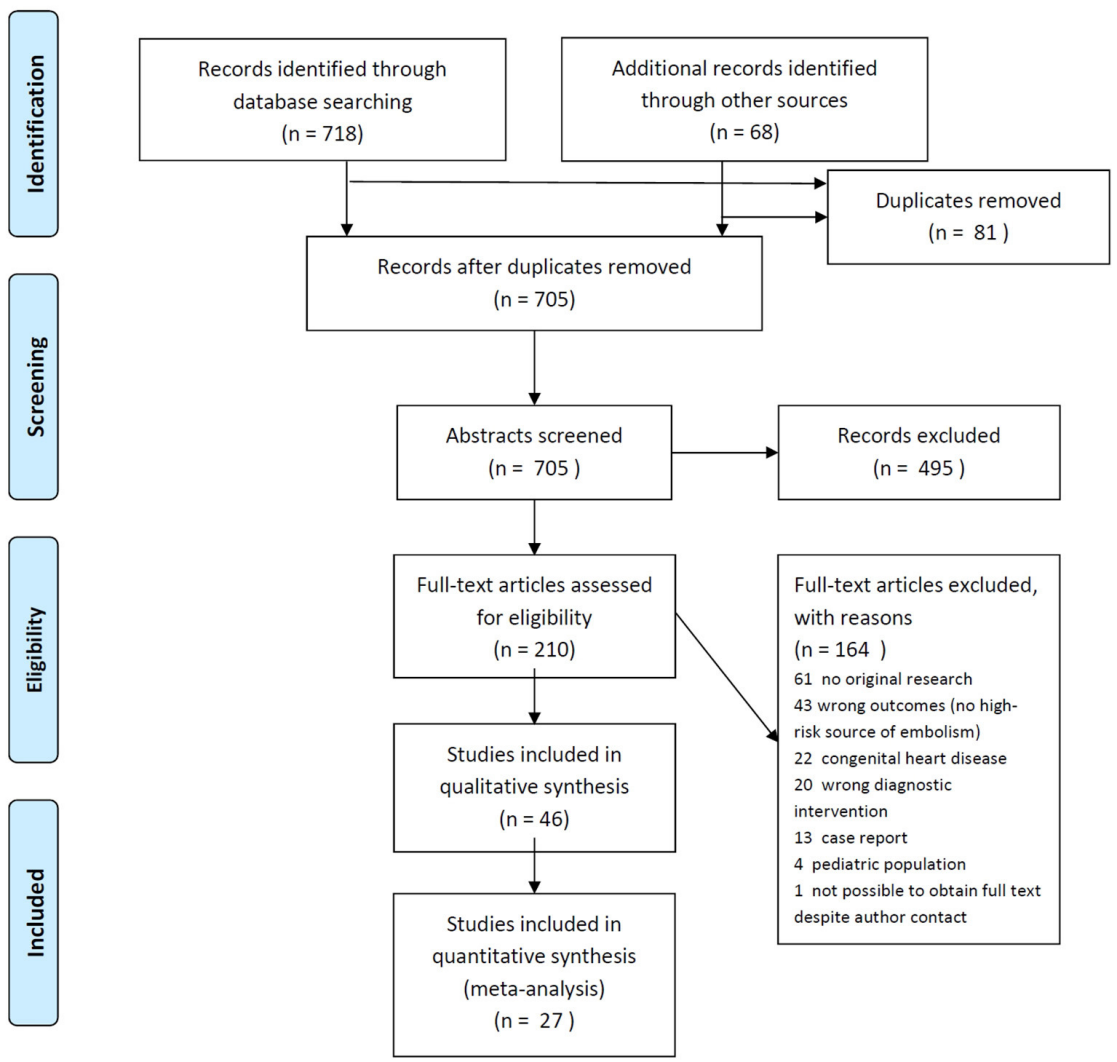
PRISMA flow diagram.

We identified 1,422 publications, of which 210 were selected for full-length review (Figure 1). Of these, 27 articles fulfilled the inclusion criteria and were included in the quantitative meta-analysis (8, 14, 21–45), further 19 studies in the qualitative report. Most studies had moderate to high risk of bias, i.e., bias relating to patient selection (non-stroke populations, highly selected stroke subgroups), to applicability, flow and timing of data acquisition (long intervals between both tests, blinding, non-random sequence), see Supplementary Material for full details.

Study characteristics of the included studies are presented in Supplementary Table 1. All were single center, academic cohort studies published between 2000 and 2020 with half of them being prospective. A total of 2,525 patients were included with study sizes from 16 to 316 patients. Eleven of the 27 studies exclusively included AIS patients, while the others mostly addressed patients with known cardiac pathologies (myocardial infarction, reduced ejection fraction, high risk for cardiac thrombus).

We report details on CMR and echocardiography methods (12/27 TEE, 11/27 TTE, 4 both; use of contrast in some studies) in Supplementary Table 1. Time interval between CMR and echocardiography ranged between 0 and 89 days and was unclear in 5/27 studies.

### Diagnostic Yield (High-Risk Sources of Embolism)

The diagnostic yield of CMR as compared to echocardiography to identify prespecified structural high-risk and medium-risk sources of embolism was similar (Table 3). Overall, persistent foramen ovale (PFO), complex aortic plaques, left ventricular (LV) and LA and LAA thrombus were the most common pathologies.

**Table 3:** Diagnostic yield of cardiovascular MRI as compared to echocardiography for detection of prespecified cardioaortic sources of embolism in studies including patients with stroke.

**Setting**	**Pathology**	***N* patients**	***N* Studies**	**Total patients with pathology on echo**	**Yield Echocardiography (95% CI)**	**Total patients with pathology on CMR**	**Yield CMR (95% CI)**	**P for comparison of CMR and Echo-cardiography**
Studies in stroke patients	Left atrial (appendage) thrombus	197	3	4	**2.0% (0.06–4.0%)**	1	**0.5% (0–1.5%)**	0.8791
	- TEE Studies	20	1	0	0%	0	0%	
	- TTE Studies	75	1	4	5.3% (0.2–10.4%)	1	1.3% (0–3.9%)	
	- Studies combination	102	1	0	0%	0	0%	
	Left ventricular thrombus	221	4	7	**3.2% (0.9–5.5%)**	11	**5.0% (2.1–7.8%)**	0.8460
	- TEE Studies	44	2	2	4.5% (0–10.7%)	2	4.5% (0–10.7%)	
	- TTE Studies	75	1	5	6.7% (1.0–12.3%)	8	10.7% (3.7–17.7%)	
	- Studies using combination	102	1	0	0%	1	1.0% (0–2.9%)	
	Valvular vegetation	289	4	6	**2.1% (0.4–3.7%)**	6	**2.1% (0.4–3.7%)**	1.0
	- TEE Studies	113	2	2	1.8% (0–4.2%)	2	1.8% (0–4.2%)	
	- TTE Studies	75	1	2	2.7% (0–6.3%)	3	4.0% (0–8.4%)	
	- Studies using combination	101	1	1	1.0% (0–2.9%)	2	2.0% (0–4.7%)	
	Non-thrombotic masses, e.g., tumor	197	3	0	**0%**	1	**0.5% (0–1.5%)**	0.9598
	- TEE Studies	20	1	0	0%	0	0%	
	- TTE Studies	75	1	0	0%	1	1.3% (0–3.9%)	
	- Studies using combination	102	1	0	0%	0	0%	
	Complex aortic plaques	295	5	55	**18.6% (14.2–23.1%)**	48	**16.3% (12.1–20%)**	0.5546
	- TEE Studies	118	3	50	42.4% (33.5–51.3%)	42	35.6% (27.0–44.2%)	
	- TTE Studies	75	1	2	2.7% (0–6.3%)	0	0%	
	- Studies using combination	102	1	3	2.9% (0–6.2%)	6	5.9% (1.3–10.4%)	
	Persistent foramen ovale (all TEE)	246	5	132	**53.7% (47.4–59.9%)**	72	**29.3% (23.6–35.0%)**	<0.001

*CMR, cardiovascular MRI; CI, confidence interval; P, P-value for comparison of the diagnostic yield by McNemar test. Bold represents overall findings (TEE and TTE results combined)*.

In stroke populations, the only pathology with a significant difference in diagnostic yield was PFO (CMR 29.3% vs. echocardiography 53.7%, *P* < 0.001). Some groups have reported reliable assessment with contrast-enhanced CMR using dedicated perfusion protocols (29), but overall TEE is the method of choice when PFO should be ruled out (23, 43, 44).

There was no difference in the yield of LV thrombus detection between both modalities for stroke populations. However, there was an increased yield of CMR in the 10 studies conducted in non-stroke populations (28.1 vs. 16.0%, *P* < 0.001, Supplementary Table 5). Non-thrombotic masses, i.e., cardiac tumors were overall rare in stroke populations. The same was true for valvular vegetations with only anecdotal data reporting on the comparison of both modalities (40).

### Other Potential Sources of Embolism

#### Complex Aortic Plaques

Overall, the diagnostic yield of CMR and echocardiography (almost exclusively TEE) to identify complex aortic plaque was similar. Faber et al. showed that also the measurements of plaque thickness correlated well in the aortic arch with CMR being able to identify 89% of ulcerations in the proximal aorta seen on TEE (22). Harloff et al. found that CMR in patients with cryptogenic stroke had higher diagnostic yield in the detection of aortic high risk plaques as compared to TEE (50 vs 31.1%, *p* = 0.029). In parallel, the absolute number of complex aortic plaques and thrombi detected by CMR was higher than with TEE (74 vs. 47) (45).

#### Aortic Dissection

A meta-analysis found that TEE, and CMR had equally reliable diagnostic yield for confirming or ruling out thoracic aortic dissection (46). However, TTE was inferior (47).

#### Mitral Valve Stenosis

There was strong agreement between TTE and CMR for quantification of mitral valve stenosis (limits of agreement for valve area −0.13 to 0.09 cm^2^) (48–50).

#### Cardiomyopathies

CMR was found to have higher diagnostic yield in detecting LV non-compaction cardiomyopathy as compared to TTE (51). Similarly, Fonseca et al. reported that among 132 patients with cryptogenic stroke, CMR was able to identify 7 patients with undiagnosed cardiomyopathies (4 hypertrophic, 2 restrictive, one non-compaction cardiomyopathy) (52).

#### Myocardial Infarction

CMR identified previous myocardial infarction in 13/89 (15%) of cryptogenic stroke patients, with echocardiography being able to pick up only 4/13 (31%) of those (31). Of note, only one patient had clinical manifest acute coronary syndrome and two previously known coronary artery disease.

### Other Structural Findings With Potential Management Consequence

#### LV Function

Häusler reported regional wall motion abnormalities in 4% of cryptogenic stroke patients according to echocardiography, but 12% according to CMR (31). Importantly, both systolic and diastolic dysfunction can be assessed by CMR and echocardiography, also in AIS patients (53, 54).

#### LA Volume and Function

In patients with permanent AF, correlations for TTE and CMR were fair to moderate and TTE measurements had inferior intra- and inter-observer agreement (55–57). However, CMR can be considered the gold standard for LA volume assessment (58). Atrial fibrosis was found more frequently using CMR in patients with undetermined stroke etiology as compared to other specific causes (13) and was shown to be independently associated with LAA-thrombus and spontaneous echo contrast on TEE (59). Bertelsen showed that various parameters including LA volume, LA emptying fraction or LA strain assessed by CMR feature tracking predicted risk of incident AF during follow-up using implantable loop recorders (60). However, also LAA peak emptying velocity as measured by TEE was shown to predict incident AF after cryptogenic stroke (61).

#### Feasibility and Reproducibility of CMR

Häusler et al. reported that 89/103 (86%) of AIS patients completed the entire CMR protocol including late gadolinium enhancement assessment (median duration 50 min, median 3 days after stroke). The main reasons for abortion of CMR were motion artifacts or inability to cooperate with multiple breath-holds despite the fact that such patients were already excluded at baseline (31). In contrast, 101/103 (98%) completed the TEE procedure with only one patient unable to tolerate the probe. Hellwig et al. reported that functional CMR could be obtained in 75% of AIS patients (54). In a study by Harloff et al., all 74 selected patients were able to complete a 49 min thoracic MR for aortic assessment but without cardiac-specific sequences within 1 week after stroke. However, image quality was considered low in 43%, moderate in 30% and good in 27% of sequences (45). Zahuranec et al. reported that of 28 patients planned to undergo CMR, only 20 could finally start the procedure and one additional patient stopped the scanning because of claustrophobia and in one CMR study image quality was severely affected by arrhythmia (34). Liberman et al. report that 6 out of 115 patients had uninterpretable CMR images (36). Takasugi et al. found that 42% of Asian embolic stroke of undetermined source patients were ineligible for CE-CMR due to severe renal dysfunction (58%), metal implants and other reasons (8).

Häusler et al. reported overall poor agreement for identification of relevant findings between CMR and echocardiography (*κ* = 0.24) with values slightly higher when concentrating on patients who had completed both procedures (*κ* = 0.47) (31).

#### Stroke Etiology, Prognosis

Baher et al. concluded that CMR could identify evident or possible cardiac embolic sources in 1 in 4 patients with cryptogenic AIS after a non-diagnostic TTE, however CMR led to a direct management change (initiation of anticoagulation) in only 3 of 106 patients (23). Häusler et al. reported that TEE could add substantial information for determining stroke etiology in 11 (12%) of 93 patients with otherwise cryptogenic stroke, as compared to CMR that did so in 9 (9%) patients. Those findings were consistent in 80 (86%) between the modalities (31). CMR—mainly by detection of unrecognized previous myocardial infarction—led to change of management in 6 patients (aspirin in one patient and high-dose statin therapy in five patients). Liberman et al. reported that only one single cryptogenic stroke patient out of 64 could be reclassified into another stroke etiology group by use of CMR after TEE. CMR was unable to overall reduce the percentage of cryptogenic stroke patients (36).

## Discussion

With this systematic review and meta-analysis, we provide estimates for the diagnostic yield of CMR as compared to echocardiography for the most relevant pathologies in AIS patients. Except for PFO and LV thrombus, the diagnostic yield of both modalities does not seem to differ substantially. The major problem with the available observational data is that a true gold-standard is missing for many pathologies. This also implies that the overall diagnostic yield represents a more meaningful outcome parameter than sensitivity and specificity. More importantly, the clinical benefit of the management consequences of the diagnostic findings is uncertain. Even for echocardiography, after decades of use in stroke work-up, there is no high-level evidence actually proving any benefit in functional outcome or prevention of recurrent cardiovascular events.

The dilemma of observational diagnostic studies is that the scientific standards of interventional trials are not applied, despite the fact that the tests carry significant therapeutic consequences. If there is clinical equipoise between two modalities, only a trial randomizing both diagnostic strategies could elucidate the overall clinical impact of the complex downstream management consequences. Applying the highest scientific standards to diagnostics has a huge potential to improve the value of care, eliminate redundant testing and cost savings (62). In the meantime, high-quality observational trials should try to clarify the impact of the diagnostic modality on downstream consequences such as secondary prevention.

In clinical practice, TTE is more often performed despite the fact that TEE has been shown superior in identifying most cardioaortic sources of embolism like PFO, LA thrombus and aortic arch pathologies (63–65). This is probably due to the risks and logistic constraints of TEE (66, 67). Moreover, routine echocardiography shows no clear potential sources of embolism in the great majority of stroke patients and has a low yield to detect clinically actionable findings for secondary stroke prevention (68). The low yield should be balanced against the risk of futility. Some clinicians may even consider echocardiography inappropriate in some stroke patients.

### Yield of CMR and Echocardiography for Specific Pathologies

In non-stroke settings, contrast-enhanced CMR is the gold-standard for detection of LV thrombus (30, 33, 69, 70). Contrast-enhanced transthoracic echocardiography can improve detection rate of LV thrombus, but has low sensitivity for mural or small thrombi (71). Nevertheless, echocardiography assessing left ventricular wall motion abnormalities could be a useful screening tool (71, 72). A history of myocardial infarction and low ejection fraction are useful predictors for the presence of LV thrombus (8). Importantly, CMR could have detect unrecognized myocardial infarction (73). This has therapeutic consequences since stroke patients have concomitant coronary artery disease in up to 25% (74). Additionally, CMR was found to potentially represent a useful tool differentiating true cardiac ischemia from neurogenically stunned myocardium in AIS patients with troponin elevation and select patients for urgent coronary angiography (75).

Regarding the assessment of LV function and volumes, CMR is considered the gold standard, although echocardiography remains the most widely used modality (76, 77). Since anticoagulation has no advantage over standard antiplatelet therapy in AIS patients with reduced LV function, the management consequences remain limited however (78). CMR is able to identify inherited or acquired cardiomyopathies that were missed by TTE in about 5% of patients with AIS (52). In regional areas with prevalent Chagas disease, CMR is able to identify left ventricle aneurysm and intracardiac thrombus in a relevant number of patients (79).

Overall, our findings demonstrate that CMR is clearly inferior to TEE in PFO detection. Given the benefits of closure trials, this culprit should be looked for rigorously in patients qualifying for closure. However, alternatively specific CMR protocols or transcranial contrast neurosonography should be used to detect PFO, when PFO closure would be considered and TEE is unavailable (80). CMR seems a fair alternative to TEE for LA- and LAA -thrombus identification with a pooled sensitivity and specificity of 0.80 (CI 0.63–0.91) and 0.98 (CI 0.97–0.99) (81).

CMR can be considered the gold standard for assessment of non-thrombotic masses (82). In stroke patients particularly, CMR is useful for differentiating tumors from thrombi (83).

There is paucity of data on the diagnostic yield of CMR for infectious endocarditis. So far, CMR plays not part in establishing or excluding the diagnosis of infectious endocarditis due to imaging problems of very small and highly mobile valvular structures. If there is clinical suspicion for infectious endocarditis, a TEE should be performed. For the follow-up of chronic aortic dissection, CMR seems a suitable imaging modality (84). Yet, in acute aortic dissection, the time advantage favors CT-angiography.

Overall, CMR seems to have reasonable yield in detection and monitoring of mitral valve stenosis. However, according to the most recent guidelines, echocardiography is the preferred method for diagnosing mitral stenosis (85).

### Management Consequences, Feasibility, and Reproducibility

Apart from high-risk sources of embolism, several structural findings might have implications for AIS patients: LA size and fibrosis increase the risk for paroxysmal AF, stroke and can potentially optimize patient selection for long-term rhythm monitoring strategies or trials of anticoagulation in patients with atrial disease without proven AF (12, 60, 86, 87). However, similar findings can also be assessed using echocardiography and the clinical benefit of CMR in predicting paroxysmal AF needs to be determined (88, 89).

Overall, there seem to be issues with the feasibility and reproducibility of CMR in the acute stroke workflow. Interestingly, a large number of screened patients were found to be ineligible because of kidney disease and metal implants and patient preference was not clearly in favor of CMR over TEE (34). Current CMR protocols are mostly based on sequences in the breathholding technique and most stroke patients have difficulties with cooperation in the acute phase.

The available data are insufficient to derive definite conclusions whether CMR is able to improve the classification of stroke etiology over echocardiography. At least, the evidence-based changes in secondary stroke prevention found in 6% of cryptogenic stroke patients seems modest (31). Most therapeutic changes were from moderate to high-dose statin therapy, which is anyway recommended for many patients with atherosclerotic disease and stroke due to new data (90). Other management consequences reported in historical studies are debatable nowadays (anticoagulation for PFO and complex aortic plaques). Apfalter et al. concluded in their observational non-randomized monocentric analysis, that CMR and cardiac CT might represent a valid alternative to echocardiography to predict stroke recurrence based on the presence of intracardiac thrombus, vulnerable aortic plaque, cardiac tumors, and valvular vegetation (25).

### How to Choose the Best Imaging Modality Until Better Evidence Is Available?

A score has been developed to identify patients with high yield of additional TEE after TTE (91), but consensus is lacking. Different recommendations for patient selection have been made without a high evidence level showing improved clinical outcomes according to such an approach (19). The question is whether a “one size fits all” approach benefits patients or a sophisticated individualized decision is more beneficial. ESUS represents a subcategory of ischemic cryptogenic stroke with non-lacunar imaging features in stroke patients without an immediately identifiable cause such as large vessel disease, significant intracranial atherosclerosis, or high-risk cardioembolic source. However, it is unlikely that cardiac work-up should differ according to these categories since findings are expected to be similar due to the shared risk factors.

Currently, CMR represents a diagnostic option in experienced centers and cooperative patients with embolic stroke in the case of non-discriminative echocardiography or a high suspicion for intracardiac thrombus or concomitant coronary artery disease. Furthermore, CMR might be used for work-up of a cardiac mass seen on an echocardiography as well as in patients who decline TEE or in case of medical reasons against TEE. If CMR is performed, it is important to include all relevant sequences for reliable detection of cardio-aortic sources of embolism and protocols have been published for this purpose (92). Cardiac CT might be a promising alternative to TEE, at least for the detection of cardiac thrombi and aortic pathologies in AIS patients (93, 94). However, due to missing data on outcome events this approach—similar to CMR—requires further study in prospective randomized diagnostic trials.

The difference in contrast media might also inform the choice of imaging modality. For CMR, most often gadolinium-based contrast agents are used. Their main limitations include impaired renal function with nephrogenic systemic fibrosis in rare cases, allergic reactions as well as pregnancy. However, the risk for nephrogenic systemic fibrosis is minimal for non-ionic cyclic gadolinium-based contrast medium even in patients with severe renal impairment. The long-term health effects of an uptake of small proportions of unchelated gadolinium into the brain and other body parts are currently unknown (95). For cardiac CT, contrast quantity is always high limiting its use in patients with impaired renal function (especially in patients taking metformin). Additionally, irradiation makes CT impossible in pregnant women and problematic in younger patients (96).

There are big variations of costs and durations of each procedure according to the procotol, use of contrast agent and health care system, however, in most settings, TTE will be the least expensive, followed by TEE, cardiac CT and CMR with a duration of roughly 1 h for most exams.

### Limitations

The studies included in the meta-analysis represent heterogeneous, highly selected populations. Risk of bias was high for most items. Most of the cardio-aortic sources of embolism have no clear definition and data are lacking to support evidence-based changes in management based on those findings. Most studies used TTE, which hampers to draw conclusions for TEE, however this also reflects clinical practice with low rates of TEE use. Additionally, due to the restriction to studies using both examinations, our findings only apply to centers that have access and the capacity to perform both modalities. In clinical practice, indications for selection of work-up modality will be heterogeneous and differ among different institutions and clinical settings, hence questioning the generalizability of the findings. Complications of TEE and CMR (especially use of contrast media) were not reported by most of the studies and hence only discussed cursorily. The time required and cost of the various diagnostic methods are not mentioned since the focus of this study was on diagnostic findings and management.

## Conclusions

Echocardiography and CMR seem to have similar diagnostic yield for most cardioaortic sources of embolism except persistent foramen ovale (higher yield in echocardiography) and left ventricular thrombus (higher yield in CMR). In order to be used routinely in clinical practice, CMR has to prove that it adds value to identification of high-risk sources of embolism, is feasible in the AIS setting, and—most importantly—improves clinical outcome events by prompting evidence-based downstream therapeutic consequences in a cost-effective manner. The next generation of stroke researchers should apply the quality standards of interventional trials using randomization and clinical outcomes to the diagnostic tests used in stroke comparing the available diagnostic imaging modalities (TTE, TEE, cardiac CT, CMR). Until this effort has been made, echocardiography will remain the cornerstone of cardiac workup. CMR represents an alternative in experienced centers, but faces problems of feasibility.

## Data Availability Statement

The original contributions presented in the study are included in the article/Supplementary Material, further inquiries can be directed to the corresponding author/s.

## Author Contributions

TM, AE, CS, and SJ contributed to conception and design of the study. TM and AE performed literature search, data extraction, and wrote the first draft of the manuscript. TM performed the statistical analysis. All authors contributed to manuscript revision, read, and approved the submitted version.

## Conflict of Interest

The authors declare that the research was conducted in the absence of any commercial or financial relationships that could be construed as a potential conflict of interest. The reviewer BM declared a former shared affiliation, with no collaboration, with the authors to the handling Editor.

## Publisher's Note

All claims expressed in this article are solely those of the authors and do not necessarily represent those of their affiliated organizations, or those of the publisher, the editors and the reviewers. Any product that may be evaluated in this article, or claim that may be made by its manufacturer, is not guaranteed or endorsed by the publisher.
