# Supplementary material for: Cardiovascular MRI Compared to Echocardiography to Identify Cardioaortic Sources of Ischemic Stroke: A Systematic Review and Meta-Analysis
Source: Front Neurol. 2021 Jul 30;12:699838. doi: 10.3389/fneur.2021.699838 (PMC8362907; doi:10.3389/fneur.2021.699838)
Supplement: Supplementary file 1 [file Table_1.docx]

**Cardiac magnetic resonance imaging as compared to echocardiography to identify high-risk cardioaortic sources of acute ischemic stroke – a systematic review and meta-analysis**

**Authors:** Thomas R Meinel, MD^a^, Angela Eggimann^a^, Kristina Brignoli^a^, Kerstin Wustmann, MD^b^, Eric Buffle, MD^b^, Felix G Meinel, MD^c^, Jan F Scheitz, MD^d^, Christian H Nolte, MD^d^, Christoph Gräni, MD PhD^b^, Urs Fischer, MD MSc^a^, Johannes Kaesmacher, MD^e^, David J Seiffge, MD^a^, Christian Seiler, MD^b^ & Simon Jung, MD^a^

**Online Supplemental Material**

| **Study** | **Country** | **Year** | **Risk source of embolism** | **No of patients** | **Inclusion criteria** | **Exclusion criteria** | **Data collection e.g. pro/retrospective** | **Target condition** | **Cardiac MRI** | **Echocardiography** |
| --- | --- | --- | --- | --- | --- | --- | --- | --- | --- | --- |
| **Mohammad**^1^ | **Saudi-Arabia** | **2020** | multiple | 24 | cryptogenic AIS (24h monitoring) | not specified | prospective | not specified | non-contrast |  |
| **Faber**^2^ | **USA** | **2013** | moderate | 22 | cryptogenic stroke patients age 18 or above, with stroke or TIA in the last 90 days, AA atheroma detected and measured on a clinically indicated TEE, and the ability to consent. | MRI contraindications, size or claustrophobia, prior history of decreased renal function, and irregular heart rate that could limit cMRI. | unclear | Aortic Atheroma | non-contrast | transesophageal |
| **Baher**^3^ | **USA** | **2013** | multiple | 106 | presence of stroke symptoms along with positive findings on diffusion-weighted imaging (DWI)-MRI of the brain. | evidence of intracranial hemorrhage on the initial head CT and presence of metallic prosthetics or implanted devices. | prospective | predefined endpoints | contrast | transthoracic, saline contrast |
| **Barkhausen**^4^ | **Germany** | **2002** | high | 24 | referred for suspected cardiac thrombus | unclear | prospective | cardiac thrombus | ECG-triggered contrast-enhanced cardiac MR | TTE in all, TEE in 11/24 |
| **Apfalter**^5^ | **USA** | **2020** | high | 75 | cardioembolic stroke patients, CMR performed | non-cardioembolic stroke | retrospective | thrombus, vulnerable aortic plaque, valvular vegetation or intracardiac tumor | contrast, breath-hold | parasternal long and short-axis imaging, including all valves and Doppler assessments, bubble-agitated saline study was performed in patients ≤55 years |
| **Kitkungvan**^6^ | **USA** | **2016** | high | 261 | patients referred for pulmonary vein isolation | TEE more than 7 days from CMR, others unclear | retrospective | LA/LAA thrombus | 1.5 or 3T, contrast | TEE in all |
| **Rathi** ^7^ | **USA** | **2013** | high | 97 | patients referred for pulmonary vein isolation, atrial fibrillation | more than 1 month. Patients with valve prosthesis, pacemakers, claustrophobia, and unsuccessful TEE examination | retrospective | LA/LAA thrombus | 1.5T, contrast | TEE in all |
| **Ohyama**^8^ | **Japan** | **2003** | high | 50 | nonrheumatic continuous AF and a history of cardioembolic stroke | implanted metallic devices (n⫽3; artificial pacemaker [3 of 3] or prosthetic valve [0 of 3]), claustrophobia (n⫽1), unsuccessful TEE probe insertion (n⫽11), and refusal to grant written informed consent (n⫽24). The | unclear | LA/LAA thrombus | 1.5T, contrast | TEE in all |
| **Mohrs** ^9^ | **USA** | **2006** | high | 25 | referred for TEE on the basis of chronic atrial fibrillation (n = 19) or a his- tory of stroke in patients with paroxysmal atrial fi- brillation (n = 2) or sinus rhythm (n | contraindications to MRI, such as pacemak- ers, defibrillators, and severe claustrophobia | prospective | LA/LAA thrombus | 1.5T, contrast | TEE in all |
| **Delewi**^10^ | **The Netherlands** | **2012** | high | 194 | first STEMI | unsuccessful PCI, hemodynamic instabil- ity, elevation of creatine kinase (CK) or CK-myocardial band <10 times the local laboratory upper limit of normal, and contraindica- tions for CMR. | prospective | LV thrombus | 1.5T, contrast | TTE in all |
| **Häusler**^11^ | **Germany** | **2017** | high | 103 | Cryptogenic stroke, adult, scheduled for TEE | Unable to cooperate with multiple breath-holds of a few seconds Glomerular, renal failure, pregnancy or lactation | prospective | multiple | 3T, contrast, breath-hold | all TEE, 79 also TTE |
| **Mollet**^12^ | **Belgium** | **2002** | high | 57 | myocardial infarction (<7 days duration), chronic myocardial infarction (>7 days duration), or ischemic cardiomyopathy referred for CMR | not reported | prospective | LV thrombus | 1.5T, contrast | TTE in all |
| **Weinsaft**^13^ | **USA** | **2011** | high | 243 | impaired systolic function LVEF <=50% | none | prospective | LV thrombus | 1.5T, contrast | TTE in all, some with contrast |
| **Srichai**^14^ | **USA** | **2006** | high | 160 | Ischemic Heart Disease with a history of remote myocardial infarction > 8 weeks | | retrospective | LV thrombus | 1.5T, contrast | TTE and TEE in all |
| **Zahuranec**^15^ | **USA** | **2011** | multiple | 20 | nonlacunar ischemic stroke | contraindications for CMR and concurrent stroke etiologies | prospective | multiple, feasibility | 1.5T, contrast | TEE in all |
| **Leddet**^16^ | **France** | **2010** | high | 56 | suspicion of intracardiac thrombus or mass on echocardiography or levocardiography | not reported | retrospective | intraventricular thrombus | 1 or 1.5T, contrast | TTE in all |
| **Liberman**^17^ | **USA** | **2017** | multiple | 93 | ischemic stroke or TIA and CMR as well as TEE performed | not reported | retrospective | multiple | 1.5 or 3T, contrast | TEE in all |
| **Joshi**^18^ | **Canada** | **2012** | high | 52 | ICD candidates | coronary revascularization or cardiac resynchronization therapy between echocardiography and CMR examinations were excluded. | retrospective | LV thrombus | 1.5T, contrast | TTE in all |
| **Takasugi**^19^ | **Japan** | **2017** | high | 60 | history of MI, low EF | contraindications for CMR, palliative care, renal failure | prospective | LV thrombus | 1.5T, contrast | TTE in all |
| **Staab**^20^ | **Germany** | **2014** | multiple | 171 | history of CAD | not reported | retrospective | LV thrombus, cardiac mass | 1.5T, contrast | TTE in all |
| **Bruder**^21^ | **Germany** | **2005** | high | 82 | history of MI with revascularization | not reported | prospective | LV thrombus | 1.5T, contrast | TTE in all |
| **Dursun**^22^ | **Turkey** | **2015** | high | 16 | preliminary diagnosis of infectious endocarditis by clinical, laboratory, and echocardiographic findings | not reported | prospective | Valvular vegetation | 1.5T, contrast | 16 TTE, 12 TEE |
| **Hooks**^23^ | **USA** | **2020** | high | 119 | consecutive adult patients diagnosed with LV thrombus after referral for a clinical CMR | not reported | retrospective | LV thrombus | 1.5T, contrast | TTE in all, contrast if necessary |
| **Patel**^24^ | **USA** | **2016** | high | 44 | patients who underwent CMR and echocardiography evaluation of a cardiac mass with subsequent histopathologic diagnosis | not reported | retrospective | LV mass/tumor | 1.5T or 3T, contrast | TTE or TEE |
| **Hamilton-Craig**^25^ | **Italy** | **2011** | Moderate | 25 | cryptogenic ischaemic stroke | not reported | retrospective | PFO | 1.5T, contrast | TEE in all |
| **Nusser**^26^ | **Germany** | **2006** | moderate | 75 | cryptogenic ischaemic stroke | not reported | retrospective | PFO | 1.5T, contrast | TEE in all |
| **Harloff**^27^ | **Germany** | **2008** | moderate | 74 | age 17–85 years, acute cryptogenic brain ischaemia, performance of TOE examination, no contraindications to 3 T MRI examination. | not reported | prospective | Aortic Atheroma | 3T, contrast | TEE in all |

**Supplement Table 1 – Details on included studies**

| **Study** | **Year** | **No of exclusions** | **No of findings/total analysed** | **risk of bias** | | | | **Concerns about applicability** | | |
| --- | --- | --- | --- | --- | --- | --- | --- | --- | --- | --- |
|  |  |  |  | Patient selection | Index Test | Reference Standard | Flow and Timing | Patient selection | Index Test | Reference standard |
| **Mohammad** | **2020** | 3 | 13 | high | high | high | high | low | low | high |
| **Faber** | **2013** | unclear | NA | high | high | high | high | low | low |  |
| **Baher** | **2013** | 35 | 7 | high | high | high | high | high | low | low |
| **Barkhausen** | **2002** | unclear | 15 | high | high | high | high | high | low | low |
| **Apfalter** | **2020** | 382 | 14 | high | moderate | moderate | moderate | high | low | low |
| **Kitkungvan** | **2016** | not reported | 9 | low | low | low | moderate | high | low | low |
| **Rathi** | **2013** | not reported | 2 | high | low | low | moderate | high | low | low |
| **Ohyama** | **2003** | 0 | 16 | high | low | low | low | high | low | low |
| **Mohrs** | **2006** | 0 | 19 | high | low | low | moderate | high | low | low |
| **Delewi** | **2012** | 6 | 9 | low | low | low | low | high | low | high |
| **Häusler** | **2017** | 1 | 13 | low | low | low | low | low | low | low |
| **Mollet** | **2002** | 0 | 12 | high | low | low | low | high | low | low |
| **Weinsaft** | **2011** | 298 | 28 | high | low | high | high | high | low | high |
| **Srichai** | **2006** | 199 | 48 | high | low | low | high | high | low | low |
| **Zahuranec** | **2011** | 109 | 6 | high | low | low | high | high | low | low |
| **Leddet** | **2010** | not reported | 26 | high | low | low | high | high | low | high |
| **Liberman** | **2017** | not reported | NA | low | low | low | low | low | low | low |
| **Joshi** | **2012** | 0 | 6 | high | low | low | high | high | low | low |
| **Takasugi** | **2017** | 45 | 12 | high | low | low | moderate | low | low | low |
| **Staab** | **2014** | not reported | 70 | high | low | low | high | high | low | low |
| **Bruder** | **2005** | not reported | 35 | high | low | low | high | high | low | low |
| **Dursun** | **2015** | not reported | 15 | high | low | low | high | high | low | low |
| **Hooks** | **2020** | not reported | 119 | high | low | low | high | high | low | low |
| **Patel** | **2016** | not reported | 44 | high | high | high | high | high | high | high |
| **Hamilton-Craig** | **2011** | 0 | 16 | low | low | low | moderate | low | low | low |
| **Nusser** | **2006** | 0 | 75 | high | low | low | high | high | low | low |
| **Harloff** | **2008** | 0 | 37 | high | low | low | high | high | low | low |

**Supplement Table 2 – Risk of bias, concerns about applicability**

| **Study** | **Year** | **PARTICIPANT SELECTION (1)  - RISK OF BIAS** | | | | | **PARTICIPANT SELECTION (1)  - CONCERNS REGARDING APPLICABILITY** | | |
| --- | --- | --- | --- | --- | --- | --- | --- | --- | --- |
|  |  | **1) Was a consecutive or random sample of participants or images  enrolled?** | **2) Was a case - control design avoided?** | **3) Did the study avoid inappropriate exclusions, e.g. · ‘difficult to diagnose’ lesions not excluded · lesions not excluded on basis of disagreement between  evaluators** | **Could the selection of participants have introduced bias?** | **1) Are the included patients and chosen study setting appropriate to  answer the review question,  i.e. are the study results generalisable?** | | **Is there concern that the included participants do not match the review  question?** |  |
| **Mohammad** | **2020** | yes | yes | no | yes | yes | | no |  |
| **Faber** | **2013** | unclear | unclear | no | yes | yes | | no |  |
| **Baher** | **2013** | yes | yes | no | yes | no | | yes |  |
| **Barkhausen** | **2002** | no | yes | no | yes | no | | yes |  |
| **Apfalter** | **2020** | no | yes | no | yes | no | | yes |  |
| **Kitkungvan** | **2016** | yes | yes | unclear | yes | no | | yes |  |
| **Rathi** | **2013** | yes | yes | no | yes | no | | yes |  |
| **Ohyama** | **2003** | yes | yes | no | yes | no | | yes |  |
| **Mohrs** | **2006** | yes | yes | no | yes | no | | yes |  |
| **Delewi** | **2012** | yes | yes | no | yes | no | | yes |  |
| **Häusler** | **2017** | yes | yes | no | yes | yes | | no |  |
| **Mollet** | **2002** | yes | yes | no | yes | no | | yes |  |
| **Weinsaft** | **2011** | yes | yes | no | yes | no | | yes |  |
| **Srichai** | **2006** | yes | yes | no | yes | no | | yes |  |
| **Zahuranec** | **2011** | yes | yes | no | yes | no | | no |  |
| **Leddet** | **2010** | yes | yes | no | yes | no | | yes |  |
| **Liberman** | **2017** | no | yes | no | yes | yes | | no |  |
| **Joshi** | **2012** | yes | yes | yes | yes | no | | yes |  |
| **Takasugi** | **2017** | yes | yes | no | yes | yes | | no (ESUS subgroup) |  |
| **Staab** | **2014** | yes | yes | yes | yes | no | | yes |  |
| **Bruder** | **2005** | yes | yes | no | yes | no | | yes |  |
| **Dursun** | **2015** | no | no | no | yes | no | | yes |  |
| **Hooks** | **2020** | no | no | no | yes | no | | yes |  |
| **Patel** | **2016** | no | no | no | yes | no | | yes |  |
| **Hamilton-Craig** | **2011** | yes | yes | yes | yes | yes | | no |  |
| **Nusser** | **2006** | no | yes | no | yes | no | | yes |  |
| **Harloff** | **2008** | no | yes | no | yes | no | | yes |  |

**Supplement Table 3 – Risk of bias: participant selection**

| **Study** | **Year** | **INDEX TEST  (2)  - CONCERN ABOUT APPLICABILITY** | | **REFERENCE STANDARD (3)  - RISK OF BIAS** | | **REFERENCE STANDARD (3)  - CONCERN ABOUT APPLICABILITY** | **FLOW AND TIMING (4): RISK OF BIAS** | | | | |
| --- | --- | --- | --- | --- | --- | --- | --- | --- | --- | --- | --- |
|  |  | 1) Was the test applied and interpreted in a clinically applicable  manner | 2) Were thresholds or criteria for diagnosis reported in sufficient  detail to allow replication? | 1) Was the CMR/echo protocol appropriate? | 2.) blinding between both tests? | Is there concern that the target condition as defined by the reference  standard does not match the review question? | 1) Was there an appropriate interval between index test and  reference standard? | 2) Did all participants receive the same reference standard? | 3) Were all participants included in the analysis? | Could the participant flow have introduced bias? |  |
| **Mohammad** | **2020** | yes | no | no | yes | no | yes (Both the TEE and CMRI were arranged and performed within 1 week from the stroke onset. The) | yes | yes | yes |  |
| **Faber** | **2013** | yes | yes | yes | no | no | no | yes | unclear | yes |  |
| **Baher** | **2013** | yes | no | yes | yes | yes | unclear | yes | no | yes |  |
| **Barkhausen** | **2002** | yes | no | yes | yes | no | unclear | no | yes | yes |  |
| **Apfalter** | **2020** | yes | yes | yes | yes | no | no (three days difference) | yes | no | yes |  |
| **Kitkungvan** | **2016** | yes | yes | yes | yes | no | no (seven days difference allowed) | yes | yes | yes |  |
| **Rathi** | **2013** | yes | no | yes | yes | no | no (up to 1 month) | yes | no | yes |  |
| **Ohyama** | **2003** | yes | yes | yes | yes | no | yes (bot the same day) | yes | no | no |  |
| **Mohrs** | **2006** | yes | yes | yes | yes | no | yes (at a time delay of 1 ± 2 days) | yes | yes | yes |  |
| **Delewi** | **2012** | yes | yes | yes | yes | no | yes | yes | no | no |  |
| **Häusler** | **2017** | yes | yes | yes | yes | no | yes | no | no | no |  |
| **Mollet** | **2002** | yes | no | yes | yes | no | yes (within 1 day) | yes | yes | no |  |
| **Weinsaft** | **2011** | yes | yes | yes | yes | no | no (within one week) | no | no | yes |  |
| **Srichai** | **2006** | yes | yes | yes | no | yes | no (within 30 days) | no | no | yes |  |
| **Zahuranec** | **2011** | yes | yes | yes | yes | no | no (within 6 days median) | yes | no | yes |  |
| **Leddet** | **2010** | yes | yes | yes | unclear | no | no (not reported) | no | no | yes |  |
| **Liberman** | **2017** | yes | no | yes | yes | yes | yes (one day (interquartile range: 0–3)) | no | yes | yes |  |
| **Joshi** | **2012** | yes | no | yes | yes | no | no (within 30 days) | yes | yes | yes |  |
| **Takasugi** | **2017** | yes | yes | yes | yes | no | no (3±2 and 5±2 days after admission) | yes | yes | yes |  |
| **Staab** | **2014** |  | yes | yes | yes | no | no (18 ± 7 days) | yes | yes | yes |  |
| **Bruder** | **2005** | yes | no | yes | yes | no | not reported | yes | yes | yes |  |
| **Dursun** | **2015** | yes | no | yes | no | yes | not reported | no | yes | yes |  |
| **Hooks** | **2020** | yes | yes | yes | yes | no | no (within 10 days) | yes | yes | yes |  |
| **Patel** | **2016** | yes | yes | yes | no | yes | no (13 +/- 34 days) | no | no | yes |  |
| **Hamilton-Craig** | **2011** | yes | yes | yes | yes | no | yes (within 1 week) | yes | yes | yes |  |
| **Nusser** | **2006** | yes | yes | yes | yes | no | no (between 0 and 89 days before closure) | yes | yes | yes |  |
| **Harloff** | **2008** | yes | yes | yes | yes | yes (also descending aortic plaques) | no (within 5 and 6 days (median)) | yes | yes | yes |  |

**Supplement Table 4 – Risk of bias: tests and flow**

| **Setting** | **Pathology** | **N patients** | **N Studies** | **Total patients with pathology on echo** | **Yield Echocardiography (95% CI)** | **Total patients with pathology on CMR** | **Yield CMR (95% CI)** | **P for comparison of CMR and Echo-cardiography** |
| --- | --- | --- | --- | --- | --- | --- | --- | --- |
| **Studies in non-stroke patients** | **Left atrial (appendage) thrombus**   - Studies using TEE - Studies using combination | 456 432 24 | 5  4  1 | 43 40  3 | 9.4% (6.7 – 12.1%) 9.3% (6.5 – 12.0%)  12.5% (0 – 25.7%) | 48  45  3 | 10.5% (7.7 – 13.3%)  10.4% (7.5 – 13.3%)  12.5% (0 – 25.7%) | 0.8615 |
|  | **Left ventricular thrombus**   - Studies using TTE - Studies using combination | 1196  1042  154 | 10  9  1 | 191  180  11 | 16.0% (13.9 – 18.0%)  17.3% (15.0 – 19.6%)  7.1% (3.1 – 11.2%) | 336  294  42 | 28.1% (25.5 – 30.6%)  28.2% (25.5 – 30.9%) 27.3% (20.0 – 34.3%) | 0.0008 |
|  | **Valvular vegetation** (1 study using combination) | 16 | 1 | 11 | 68.8% (46.0 – 91.5%) | 15 | 93.8% (81.9 – 100%) | 0.2188 |
|  | **Non-thrombotic masses, e.g. tumor**   - Studies using TTE - Studies using combination | 215  171  44 | 2  1  1 | 59  15  44 | 27.4% (21.5 – 33.4%)  8.8% (4.5 – 13.0%)  100% | 51  12  39 | 23.7% (18.0 – 29.4%)  7.0% (3.2 – 10.8%)  88.6% (79.3 – 98.0%) | 0.6547 |
|  | **Persistent foramen ovale** (1 study using TEE) | 20 | 1 | 15 | 75% (56.0 – 94.0%) | 15 | 75% (56.0 – 94.0%) | 1.000 |

**Supplement Table 5 – Diagnostic yield of cardiac MRI as compared to echocardiography for detection of prespecified cardioaortic sources of embolism in non-stroke populations.**CMR: cardiac MRI; CI: confidence interval; P: P-value for comparison of the diagnostic yield by McNemar test.


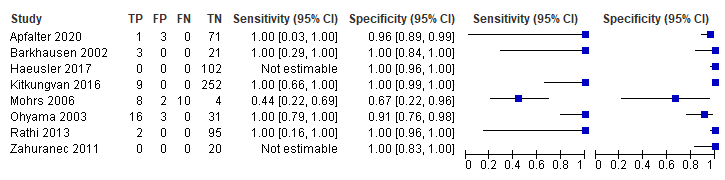


**Supplement Figure 1 - Forest plot of sensitivity and specificity of CMR (index test) as compared to echocardiography (reference standard) for the detection of left atrial (appendage) thrombus in all populations**

**
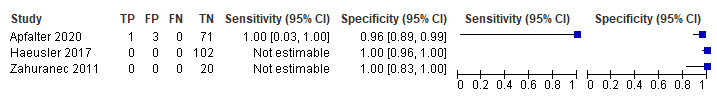
**

**Supplement Figure 2 - Forest plot of sensitivity and specificity of CMR (index test) as compared to echocardiography (reference standard) for the detection of left atrial (appendage) thrombus in stroke patient populations**

**
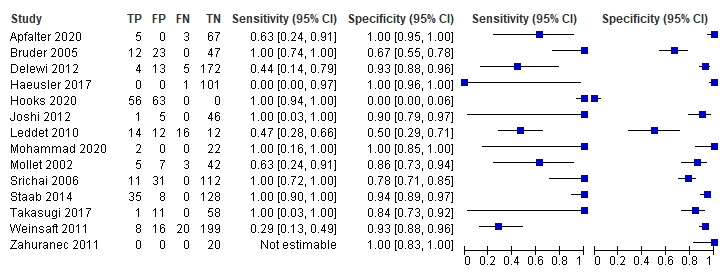
**

**Supplement Figure 3 - Forest plot of sensitivity and specificity of CMR (index test) as compared to echocardiography (reference standard) for the detection of left ventricular thrombus in all populations**

**
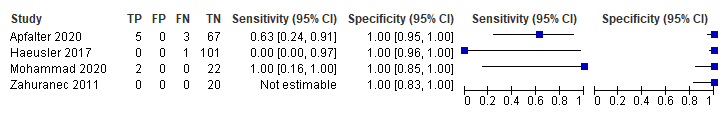
**

**Supplement Figure 4 - Forest plot of sensitivity and specificity of CMR (index test) as compared to echocardiography (reference standard) for the detection of left ventricular thrombus in stroke patient populations**

**
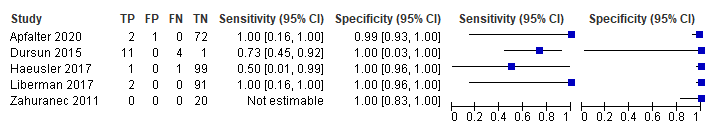
**

**Supplement Figure 5 - Forest plot of sensitivity and specificity of CMR (index test) as compared to echocardiography (reference standard) for the detection of valve vegetations in all populations**

**
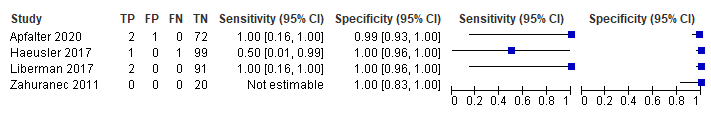
**

**Supplement Figure 6 - Forest plot of sensitivity and specificity of CMR (index test) as compared to echocardiography (reference standard) for the detection of valve vegetations in stroke patient populations**

**
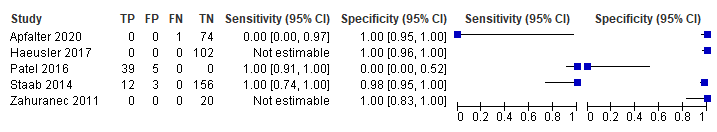
**

**Supplement Figure 7 - Forest plot of sensitivity and specificity of CMR (index test) as compared to echocardiography (reference standard) for the detection of non-thrombotic masses (e.g. cardiac tumor) in all populations**

**
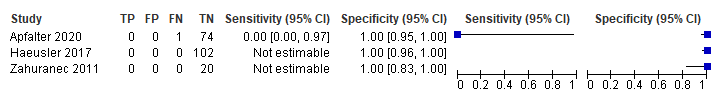
**

**Supplement Figure 8 - Forest plot of sensitivity and specificity of CMR (index test) as compared to echocardiography (reference standard) for the detection of non-thrombotic masses (e.g. cardiac tumor) in stroke patient populations**

**
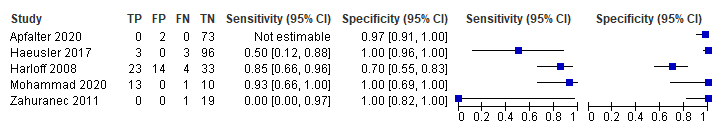
**

**Supplement Figure 9 - Forest plot of sensitivity and specificity of CMR (index test) as compared to echocardiography (reference standard) for the detection of complex aortic plaques in stroke patient populations**

**
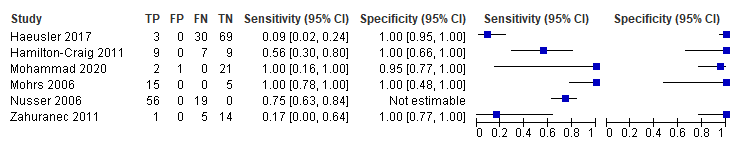
**

**Supplement Figure 10 - Forest plot of sensitivity and specificity of CMR (index test) as compared to echocardiography (reference standard) for the detection of persistent foramen ovale in all patient populations**

**
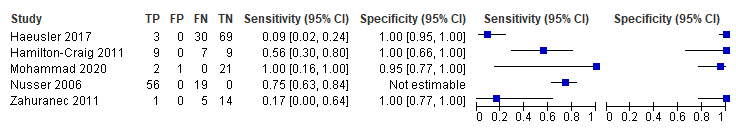
**

**Supplement Figure 11 - Forest plot of sensitivity and specificity of CMR (index test) as compared to echocardiography (reference standard) for the detection of persistent foramen ovale in stroke patient populations**

**References**

51. Diwadkar S, Nallamshetty L, Rojas C, Athienitis A, Declue C, Cox C, et al. Echocardiography fails to detect left ventricular noncompaction in a cohort of patients with noncompaction on cardiac magnetic resonance imaging. Clin Cardiol. 2017;40(6):364-369. doi:10.1002/clc.22669

52. Fonseca AC, Marto JP, Pimenta D, Guimarães T, Alves PN, Inácio N, et al. Undetermined stroke genesis and hidden cardiomyopathies determined by cardiac magnetic resonance. Neurology. 2020;94(1):e107-e113. doi:10.1212/WNL.0000000000008698

53. Schaafs L-A, Wyschkon S, Elgeti M, Nagel SN, Knebel F, Steffen IG, et al. Diagnosis of Left Ventricular Diastolic Dysfunction Using Cardiac Magnetic Resonance Imaging: Comparison of Volume-Time Curves Derived from Long- and Short-Axis Cine Steady-State Free Precession Datasets. RöFo - Fortschritte auf dem Gebiet der Röntgenstrahlen und der Bildgeb Verfahren. Published online 2020. doi:10.1055/a-1108-1892

54. Hellwig S, Grittner U, Elgeti M, Wyschkon S, Nagel SN, Fiebach JB, et al. Evaluation of left ventricular function in patients with acute ischaemic stroke using cine cardiovascular magnetic resonance imaging. ESC Hear Fail. Published online 2020. doi:10.1002/ehf2.12833

55. Agner BFR, Kühl JT, Linde JJ, Kofoed KF, Åkeson P, Rasmussen B V., et al. Assessment of left atrial volume and function in patients with permanent atrial fibrillation: Comparison of cardiac magnetic resonance imaging, 320-slice multi-detector computed tomography, and transthoracic echocardiography. Eur Heart J Cardiovasc Imaging. 2014;15(5):532-540. doi:10.1093/ehjci/jet239

56. Kühl JT, Lønborg J, Fuchs A, Andersen MJ, Vejlstrup N, Kelbæk H, et al. Assessment of left atrial volume and function: A comparative study between echocardiography, magnetic resonance imaging and multi slice computed tomography. Int J Cardiovasc Imaging. 2012;28(5):1061-1071. doi:10.1007/s10554-011-9930-2

57. Müller H, Burri H, Gentil P, Lerch R, Shah D. Measurement of left atrial volume in patients undergoing ablation for atrial fibrillation: Comparison of angiography and electro-anatomic (CARTO) mapping with real-time three-dimensional echocardiography. Europace. 2010;12(6):792-797. doi:10.1093/europace/euq031

58. Tops LF, Schalij MJ, Bax JJ. Imaging and atrial fibrillation: The role of multimodality imaging in patient evaluation and management of atrial fibrillation. Eur Heart J. 2010;31(5):542-551. doi:10.1093/eurheartj/ehq005

59. Akoum N, Fernandez G, Wilson B, Mcgann C, Kholmovski E, Marrouche N. Association of atrial fibrosis quantified using LGE-MRI with atrial appendage thrombus and spontaneous contrast on transesophageal echocardiography in patients with atrial fibrillation. J Cardiovasc Electrophysiol. 2013;24(10):1104-1109. doi:10.1111/jce.12199

60. Bertelsen L, Diederichsen SZ, Haugan KJ, Brandes A, Graff C, Krieger D, et al. Left atrial volume and function assessed by cardiac magnetic resonance imaging are markers of subclinical atrial fibrillation as detected by continuous monitoring. EP Eur. Published online 2020:1-8. doi:10.1093/europace/euaa035

61. Farinha JM, Parreira L, Marinheiro R, Fonseca M, Mesquita D, Gonçalves S, et al. A lower left atrial appendage peak emptying velocity in the acute phase of cryptogenic stroke predicts atrial fibrillation occurrence during follow-up. Echocardiography. 2019;36(10):1859-1868. doi:10.1111/echo.14478

62. Levine DA, Burke JF. Stroke imaging: Quantity, but is there quality? Med Care. 2016;54(5):423-425. doi:10.1097/MLR.0000000000000538

63. De Bruijn SFTM, Agema WRP, Lammers GJ, Van Der Wall EE, Wolterbeek R, Holman ER, et al. Transesophageal echocardiography is superior to transthoracic echocardiography in management of patients of any age with transient ischemic attack or stroke. Stroke. 2006;37(10):2531-2534. doi:10.1161/01.STR.0000241064.46659.69

64. Pearson AC, Labovitz AJ, Tatineni S, Gomez CR. Superiority of transesophageal echocardiography in detecting cardiac source of embolism in patients with cerebral ischemia of uncertain etiology. J Am Coll Cardiol. 1991;17(1):66-72. doi:10.1016/0735-1097(91)90705-E

65. Saric M, Armour AC, Arnaout MS, Chaudhry FA, Grimm RA, Kronzon I, et al. Guidelines for the Use of Echocardiography in the Evaluation of a Cardiac Source of Embolism. J Am Soc Echocardiogr. 2016;29(1):1-42. doi:10.1016/j.echo.2015.09.011

66. Hilberath JN, Oakes DA, Shernan SK, Bulwer BE, D’Ambra MN, Eltzschig HK. Safety of transesophageal echocardiography. J Am Soc Echocardiogr. 2010;23(11):1115-1127. doi:10.1016/j.echo.2010.08.013

67. Matz K. A TEE, too hard to swallow? Eur J Neurol. Published online 2020. doi:10.1111/medu.14137

68. Fralick M, Goldberg N, Rohailla S, Guo Y, Burke MJ, Lapointe-Shaw L, et al. Value of routine echocardiography in the management of stroke. Cmaj. 2019;191(31):E853-E859. doi:10.1503/cmaj.190111

69. Weinsaft JW, Kim HW, Shah DJ, Klem I, Crowley AL, Brosnan R, et al. Detection of Left Ventricular Thrombus by Delayed-Enhancement Cardiovascular Magnetic Resonance. Prevalence and Markers in Patients With Systolic Dysfunction. J Am Coll Cardiol. 2008;52(2):148-157. doi:10.1016/j.jacc.2008.03.041

70. Roifman I, Connelly KA, Wright GA, Wijeysundera HC. Echocardiography vs Cardiac Magnetic Resonance Imagingfor the Diagnosis of Left Ventricular Thrombus: A Systematic Review. Can J Cardiol. 2015;31(6):785-791. doi:10.1016/j.cjca.2015.01.011

71. Weinsaft JW, Kim RJ, Ross M, Krauser D, Manoushagian S, LaBounty TM, et al. Contrast-Enhanced Anatomic Imaging as Compared to Contrast-Enhanced Tissue Characterization for Detection of Left Ventricular Thrombus. JACC Cardiovasc Imaging. 2009;2(8):969-979. doi:10.1016/j.jcmg.2009.03.017

72. Weinsaft JW, Kim J, Medicherla CB, Ma CL, Codella NCF, Kukar N, et al. Echocardiographic Algorithm for Post-Myocardial Infarction LV Thrombus A Gatekeeper for Thrombus Evaluation by Delayed Enhancement CMR. JACC Cardiovasc Imaging. 2016;9(5):505-515. doi:10.1016/j.jcmg.2015.06.017

73. Merkler AE, Sigurdsson S, Eiriksdottir G, Safford MM, Phillips CL, Iadecola C, et al. Association between Unrecognized Myocardial Infarction and Cerebral Infarction on Magnetic Resonance Imaging. JAMA Neurol. 2019;76(8):956-961. doi:10.1001/jamaneurol.2019.1226

74. Adams RJ, Chimowitz MI, Alpert JS, Awad IA, Cerqueria MD, Fayad P, et al. AHA / ASA Scientific Statement Coronary Risk Evaluation in Patients With Transient Ischemic Attack and Ischemic Stroke A Scientific Statement for Healthcare Professionals From the Stroke Council and the Council on Clinical Cardiology of the American Heart. 2003;(September):1278-1290. doi:10.1161/01.CIR.0000094444.87006.CF

75. Haeusler KG, Jensen C, Scheitz JF, Krause T, Wollboldt C, Witzenbichler B, et al. Cardiac Magnetic Resonance Imaging in Patients with Acute Ischemic Stroke and Elevated Troponin: A TRoponin ELevation in Acute Ischemic Stroke (TRELAS) Sub-Study. Cerebrovasc Dis Extra. 2019;9(1):19-24. doi:10.1159/000498864

76. Keenan NG, Pennell DJ. CMR of ventricular function. Echocardiography. 2007;24(2):185-193. doi:10.1111/j.1540-8175.2007.00375.x

77. Greupner J, Zimmermann E, Grohmann A, Dübel HP, Althoff T, Borges AC, et al. Head-to-head comparison of left ventricular function assessment with 64-row computed tomography, biplane left cineventriculography, and both 2- and 3-dimensional transthoracic echocardiography: Comparison with magnetic resonance imaging as the reference s. J Am Coll Cardiol. 2012;59(21):1897-1907. doi:10.1016/j.jacc.2012.01.046

78. Ntaios G, Vemmos K, Lip GYH. Oral anticoagulation versus antiplatelet or placebo for stroke prevention in patients with heart failure and sinus rhythm: Systematic review and meta-analysis of randomized controlled trials. International Journal of Stroke. doi:10.1177/1747493019877296

79. Neri LR, Torreão JA, Porto LM, Gonçalves BMMM, Andrade AL, Pereira CB, et al. Factors associated with abnormal cardiac magnetic resonance imaging in embolic stroke of undetermined source. Int J Stroke. 2019;14(4):NP6-NP9. doi:10.1177/1747493019840928

80. Chin SP, Ong TK, Rapaee A, Liew CK, Liew HB, Chan WL, et al. Use of non-invasive phase contrast magnetic resonance imaging for estimation of atrial septal defect size and morphology: A comparison with transesophageal echo. Cardiovasc Intervent Radiol. 2006;29(2):230-234. doi:10.1007/s00270-005-0003-6

81. Vira T, Pechlivanoglou P, Connelly K, Wijeysundera HC, Roifman I. Cardiac computed tomography and magnetic resonance imaging vs. transoesophageal echocardiography for diagnosing left atrial appendage thrombi. Europace. 2019;21(1):E1-E10. doi:10.1093/europace/euy142

82. Kumar P, Singh A, Deshmukh A, Kumar S. Cardiac MRI for the evaluation of cardiac neoplasms. Clin Radiol. 2020;75(4):241-253. doi:10.1016/j.crad.2019.11.014

83. Hong YJ, Hur J, Kim YJ, Lee HJ, Nam JE, Kim HY, et al. The usefulness of delayed contrast-enhanced cardiovascular magnetic resonance imaging in differentiating cardiac tumors from thrombi in stroke patients. Int J Cardiovasc Imaging. 2011;27 Suppl 1:89-95. doi:10.1007/s10554-011-9961-8

84. Sherrah AG, Grieve SM, Jeremy RW, Bannon PG, Vallely MP, Puranik R. MRI in Chronic Aortic Dissection: A Systematic Review and Future Directions. Front Cardiovasc Med. 2015;2. doi:10.3389/fcvm.2015.00005

85. Baumgartner H, Hung J, Bermejo J, Chambers JB, Evangelista A, Griffin BP, et al. Echocardiographic assessment of valve stenosis: EAE/ASE recommendations for clinical practice. Eur J Echocardiogr. 2009;10(1):1-25. doi:10.1093/ejechocard/jen303

86. Schnabel RB, Haeusler KG, Healey JS, Freedman B, Boriani G, Brachmann J, et al. Searching for Atrial Fibrillation Poststroke: A White Paper of the AF-SCREEN International Collaboration. Circulation. 2019;140(22):1834-1850. doi:10.1161/CIRCULATIONAHA.119.040267

87. Haeusler KG, Gröschel K, Köhrmann M, Anker SD, Brachmann J, Böhm M, et al. Expert opinion paper on atrial fibrillation detection after ischemic stroke. Clin Res Cardiol. 2018;107(10):871-880. doi:10.1007/s00392-018-1256-9

88. Johansen MC, Lin M, Nazarian S, Gottesman RF. Associations of echocardiographic features with stroke in those without atrial fibrillation. Neurology. 2019;92(9):10.1212/WNL.0000000000007002. doi:10.1212/WNL.0000000000007002

89. Jordan K, Yaghi S, Poppas A, Chang AD, Grory B Mac, Cutting S, et al. Left Atrial Volume Index Is Associated with Cardioembolic Stroke and Atrial Fibrillation Detection after Embolic Stroke of Undetermined Source. Stroke. 2019;50(8):1997-2001. doi:10.1161/STROKEAHA.119.025384

90. Amarenco P, Kim JS, Labreuche J, Charles H, Abtan J, Bejot Y, et al. A Comparison of Two LDL Cholesterol Targets after Ischemic Stroke. N Engl J Med. 2020;382(1):9-19. doi:10.1056/NEJMoa1910355

91. Charbonnel C, Jego C, Jourda F, Vinsonneau U, Garçon P, Turlotte G, et al. ADAM-C score: New risk score for predicting diagnostic yield of transesophageal echocardiography after cerebral ischemia. Echocardiography. 2018;35(8):1171-1182. doi:10.1111/echo.14010

92. Hoey ETDD, Mankad K, Al-Chalabi H, Rosa S. The emerging role of cardiovascular MRI for suspected cardioembolic stroke. Clin Radiol. 2013;68(2):107-116. doi:10.1016/j.crad.2012.07.003

93. Groeneveld NS, Guglielmi V, Leeflang MMG, Matthijs Boekholdt S, Nils Planken R, Roos YBWEM, et al. CT angiography vs echocardiography for detection of cardiac thrombi in ischemic stroke: a systematic review and meta-analysis. J Neurol. 2020;(0123456789). doi:10.1007/s00415-020-09766-8

94. Guglielmi V, Planken RN, Mihl C, Niesen S, Staals J, Coutinho JM, et al. Non-gated cardiac CT angiography for detection of cardio-aortic sources of embolism in the acute phase of ischaemic stroke. J Neurol Neurosurg Psychiatry. 2020;91(4):442-443. doi:10.1136/jnnp-2019-321923

95. Fraum TJ, Ludwig DR, Bashir MR, Fowler KJ. Gadolinium-based contrast agents: A comprehensive risk assessment. J Magn Reson Imaging. 2017;46(2):338-353. doi:10.1002/jmri.25625

96. ACR. ACR Manual On Contrast Media. Published 2021. https://www.acr.org/-/media/ACR/Files/Clinical-Resources/Contrast_Media.pdf
